# Teenager With Progressive Vision Loss After Eye Trauma

**DOI:** 10.1016/j.acepjo.2026.100352

**Published:** 2026-03-07

**Authors:** Bryce Palmer, Dharshana Krishnaprasadh

**Affiliations:** 1 Texas Tech University Health Sciences Center School of Medicine, Lubbock, Texas, USA; 2 Department of Emergency Medicine, Texas Tech University Health Sciences Center, Lubbock, Texas, USA

**Keywords:** eye trauma, globe injury, vision loss, ocular trauma

## 1 Case Presentation

**Figure 1:**
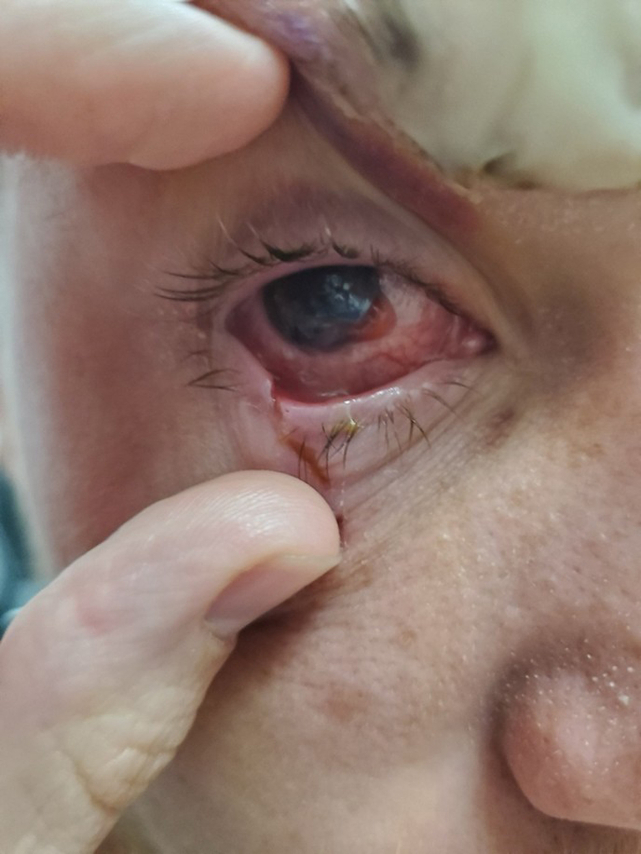
External photograph of the right eye demonstrating conjunctival injection, irregular pupil, and abnormal anterior segment appearance. Prolapsed intraocular tissue obscured clear identification of a Seidel sign.

**Figure 2:**
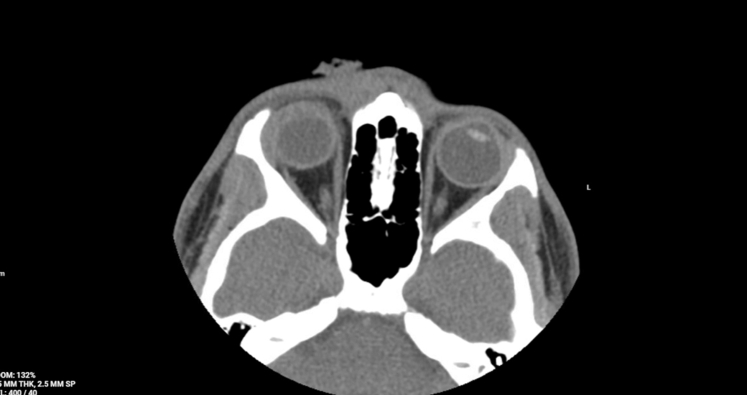
Noncontrast computed tomography scan of the orbits demonstrating absence of the right ocular lens (arrow), a concerning finding in the setting of ocular trauma.

A 17-year-old male presented to the emergency department with progressive vision loss in the right eye 5 days after being struck by a falling 2 × 4 wooden plank. His vision continued to worsen, prompting evaluation by an optometrist who referred him for emergency care. Examination revealed conjunctival injection and an opaque film overlying the cornea (Fig 1). Fluorescein examination was negative for a Seidel sign. Visual acuity was no light perception in the right eye. Computed tomography (CT) of the orbits demonstrated absence of the right ocular lens without orbital fracture (Fig 2). Slit-lamp examination revealed prolapsed lenticular material through a corneal defect. The patient was started on intravenous broad-spectrum antibiotics, ophthalmology was consulted, and he underwent surgical repair.

## 2 Diagnosis: Open Globe Injury with Prolapsed Lens

Open globe injuries are vision-threatening ophthalmologic emergencies requiring prompt recognition and operative management.^1^^,^^2^ However, classic findings such as a positive Seidel sign may be absent when prolapsed intraocular contents temporarily seal the wound, leading to false reassurance.^1^^,^^3^

CT imaging may demonstrate indirect signs of open globe injury, including lens displacement or absence,^1^^,^^2^ but CT sensitivity is imperfect, and serious injury may exist despite a well-formed globe.^2^^,^^4^ Persistent vision loss, abnormal pupil, or failure to improve after presumed corneal abrasion should prompt urgent ophthalmologic consultation. Management includes rigid eye shield placement, avoidance of increased ocular pressure, broad-spectrum intravenous antibiotics, tetanus prophylaxis, and urgent surgical repair.^1^^,^^2^^,^^5^ This case highlights the need to maintain suspicion for open globe injury even when Seidel testing is negative.

## Funding and Support

By *JACEP Open* policy, all authors are required to disclose any and all commercial, financial, and other relationships in any way related to the subject of this article as per ICMJE conflict of interest guidelines (see www.icmje.org). The authors have stated that no such relationships exist.

## Conflict of Interest

All authors have affirmed they have no conflicts of interest to declare.

## References

[1] Romaniuk V.M. (2013). Ocular trauma and other catastrophes. Emerg Med Clin North Am.

[2] Pelletier J., Koyfman A., Long B. (2023). High risk and low prevalence diseases: Open globe injury. Am J Emerg Med.

[3] Mohseni M., Blair K., Gurnani B., Bragg B.N. (2023).

[4] Rometti M., Roskoski D., Patti L. (2025). Ocular trauma following ground level fall. J Am Coll Emerg Physicians Open.

[5] Wang D., Deobhakta A. (August 1, 2020). Open-globe injury: assessment and preoperative management. *American Academy of Ophthalmology EyeNet Magazine*. https://www.aao.org/eyenet/article/open-globe-injury.

